# List-method directed forgetting: Do critical findings generalize from short to long retention intervals?

**DOI:** 10.3758/s13421-021-01192-z

**Published:** 2021-06-23

**Authors:** Magdalena Abel, Bettina Kuchler, Elisabeth Meier, Karl-Heinz T. Bäuml

**Affiliations:** Department of Experimental Psychology, Regensburg University, 93040 Regensburg, Germany

**Keywords:** Directed forgetting, List-method directed forgetting, Recall, Item recognition, Retention interval

## Abstract

People can purposefully forget information that has become irrelevant, as is demonstrated in list-method directed forgetting (LMDF). In this task, participants are cued to intentionally forget an already studied list (list 1) before encoding a second list (list 2); this induces forgetting of the first-list items. Most research on LMDF has been conducted with short retention intervals, but very recent studies indicate that such directed forgetting can be lasting. We examined in two experiments whether core findings in the LMDF literature generalize from short to long retention intervals. The focus of Experiment 1 was on the previous finding that, with short retention interval, list-2 encoding is necessary for list-1 forgetting to arise. Experiment 1 replicated the finding after a short delay of 3 min between study and test and extended it to a longer delay of 20 min. The focus of Experiment 1 was on the absence of list-1 forgetting in item recognition, previously observed after short retention interval. Experiment 1 replicated the finding after a short delay of 3 min between study and test and extended it to longer delays of 20 min and 24 h. Implications of the results for theoretical explanations of LMDF are discussed.

Most of us have stories to tell about situations in which we forgot something quite essential (e.g., the birthday of a loved one, an important deadline, or the correct answer to some test question). Consequently, we tend to see forgetting as something undesirable that just happens to us under the worst circumstances. Yet, there are also instances in which forgetting may be desirable and indeed adaptive (e.g., when it frees up mental capacities, or lets us leave a stressful experience behind). The question is: Can we forget in a targeted fashion? By now, many studies indicate that voluntary forgetting is indeed possible, and that we can, to some degree, influence what we remember and forget (e.g., Anderson & Hanslmayr, 2014; Bjork, Bjork, & Anderson, 1998; Nørby, 2015). The present work is concerned with a specific form of voluntary forgetting, namely forgetting of information that has become irrelevant and is no longer needed.

Voluntary forgetting of outdated information can be examined in the lab by means of list-method directed forgetting (LMDF; Bjork, 1970). In this task, subjects study two lists of items. Critically, after study of the first list, subjects are cued to remember the list for a later test or to forget the list, pretending that it is no longer relevant and can be forgotten. After study of the second list, memory for both lists is tested, irrespective of original cuing. The typical finding is that, compared with remember-cued participants, forget-cued participants show impaired recall of list 1 and improved recall of list 2, referred to as list-1 forgetting and list-2 enhancement (Bjork, 1989). Both list-1 forgetting and list-2 enhancement have been replicated many times, suggesting that the findings are robust and can arise across a number of experimental situations (for reviews, see Bäuml, Abel, & Kliegl, 2020; MacLeod, 1998; Sahakyan, Delaney, Foster, & Abushanab, 2013). The two effects do, however, not always arise in unison and have been suggested to be mediated by different mechanisms (e.g., Pastötter & Bäuml, 2010; Sahakyan & Delaney, 2003).

In the present work, we focused on list-1 forgetting. In particular, our goal was to examine if core findings on LMDF after short delay can be generalized to longer retention intervals. Because this questions holds direct relevance for theoretical accounts of LMDF, gaining an answer may help to better understand the mechanism(s) underlying list-1 forgetting. In the following, we will first provide an overview of theoretical accounts of LMDF and the (limited) previous work on LMDF after longer retention intervals, before diving into the details of the conducted experiments.

## Theoretical explanations of (short-delay) LMDF

One prominent account that was originally proposed for item-method directed forgetting, but later also applied to explain LMDF is the selective-rehearsal account (see Bjork, 1970). The basic idea of this account is that a remember cue after list 1 prompts participants to try to maintain the first list in memory by rehearsing it during list-2 encoding—whereas participants who receive a forget cue have no reason to do so. The supposed difference in rehearsal activities can explain the later difference in recall of list 1. Another prominent account is retrieval inhibition (Geiselman, Bjork, & Fishman, 1983). This account assumes that the forget cue initiates an inhibitory control process that impairs access to list 1 and thereby reduces recall of the first-list items. Finally, list-1 forgetting has been attributed to context change (Sahakyan & Kelley, 2002). This account suggests that the forget cue triggers a deliberate switch of mental context, such that mental context at test no longer matches the context present during study of list 1, thereby reducing recall of the first-list items.

For decades, experimental research on LMDF was almost exclusively carried out with short retention intervals between study and test, so that theoretical accounts were usually evaluated with regard to how well they explained findings arising more or less immediately after encoding. Although the selective rehearsal account can explain the basic LMDF findings, it was challenged by a number of early findings. One such finding is that list-1 forgetting after a short retention interval arises not only for intentionally studied but also for incidentally encoded materials (Geiselman et al., 1983; Sahakyan & Delaney, 2010). Because incidentally encoded materials should not be regarded as relevant for a later memory test, they should also not be subject to selective rehearsal—yet, list-1 forgetting arises for both intentionally and incidentally encoded items. Another early challenge for the selective-rehearsal account was posed by the finding that testing format plays a critical role in the effect. List-1 forgetting is usually present on recall tests but absent on recognition tests, which should not be the case if differences in rehearsal—and thus in encoding—mediated the effect (e.g., Basden, Basden, & Gargano, 1993; Geiselman et al., 1983). On the basis of these findings on short-delay LMDF, the selective-rehearsal account is often dismissed in the contemporary literature on LMDF (e.g., Bäuml, Pastötter, & Hanslmayr, 2010; Sahakyan et al., 2013; but see Delaney, Nghiem, & Waldum, 2009; Sheard & MacLeod, 2005).

In contrast, both the inhibition and the context-change accounts are consistent with a number of findings on short-delay LMDF (e.g., Bäuml et al., 2020). This holds while single studies have been interpreted as providing specific support for one account over the other (e.g., Abel & Bäuml, 2017; Sahakyan et al., 2013). Specific support for the inhibition account might for instance arise from the finding that list-1 forgetting is eliminated under divided-attention conditions. Such conditions may hamper cognitive control processes that draw on cognitive resources (Conway, Harries, Noyes, Racsmany, & Frankish, 2000; Macrae, Bodenhausen, Milne, & Ford, 1997). Furthermore, activity in the dorsolateral prefrontal cortex has been shown to be causally linked to list-1 forgetting, which may reflect the involvement of an inhibitory control process (Hanslmayr et al., 2012).

Much of the evidence supporting the context-change account consists of demonstrated parallels between LMDF and context-dependent forgetting, as it may arise, for instance, when participants are asked to engage in so-called imagination tasks (e.g., imagining what they would do if they were invisible, or walking through their childhood homes; see Sahakyan & Kelley, 2002). Such imagination tasks between the study of two lists can indeed create results for list 1 that are very similar to those created by a cue to forget the first list (e.g., Bauml & Samenieh, 2012; Pastotter & Bauml, 2007; Sahakyan & Foster, 2009; Sahakyan & Kelley, 2002). This lack of behavioral dissociations between the two forms of forgetting has often been interpreted as support for the context-change account because the account predicts that context-dependent forgetting and LMDF should be largely equivalent.

## LMDF after long retention interval

More recently, a number of studies have examined LMDF after longer delays, thus extending the previous work in the literature that had almost exclusively focused on short delays. In particular, Abel and Bäuml (2017, 2019) examined list-1 forgetting after both short (30 s or 3 min) and longer delays (20 min or 24 h). They found list-1 forgetting to be evident not only after short delays but also to persist in similar magnitude across longer delays. This pattern emerged when a free-recall test was applied to assess memory, but also when initial-letter cues for the first-list items were provided at test. Together, these findings suggest that list-1 forgetting is not a transient phenomenon but can last across a prolonged retention interval. Results consistent with these studies were recently reported by Hupbach (2018), who found list-1 forgetting in free recall after a short delay (1 min) as well as after longer delays (12 h or 24 h; for a detailed discussion of previous work on LMDF after longer delay, see Abel and Bäuml 2017).

Importantly, Abel and Bäuml (2017, 2019) assessed not only LMDF, but additionally assessed context-dependent forgetting as induced by imagination tasks after varying delays. In contrast to list-1 forgetting, context-dependent forgetting of list-1 items was only present after short delays (30 s or 3 min) and was eliminated after longer delays (20 min or 24 h). Again, this pattern emerged in both free recall and initial-letter cued recall. This finding parallels the recent finding that, in a multiple-list paradigm, the semantic generation of extra-list items between study of the lists—which, like imagination, is supposed to induce mental context change (e.g., Jang & Huber, 2008; Pastötter, Schicker, Niedernhuber, & Bäuml, 2011)—induces forgetting in immediate recall but no longer when recall is delayed by 15 min (Divis and Benjamin, 2014).

The observation that context-dependent forgetting as induced by imagination or semantic generation tasks is rather short-lived is consistent with the view that mental context fluctuates over time (e.g., Estes, 1955; McGeoch, 1932) and induction of mental context change between study of two lists enhances the contextual fluctuation. Such enhanced contextual fluctuation should increase the contextual disparity of the two lists and impair list-1 recall after short delay. However, because a prolonged retention interval will change the context sufficiently far away from the list contexts, the difference between the two list contexts may become relatively small with increasing retention interval. As a result, list-1 recall should no longer depend much on the originally induced mental context change and the forgetting of list-1 items should disappear (for details, see Divis & Benjamin, 2014; for a corresponding prediction based on the context-change account of LMDF, see Sahakyan et al., 2013, p. 161).

The results reported by Abel and Bäuml (2017, 2019) dissociate LMDF and context-dependent forgetting as induced by imagination tasks. They thus challenge the context-change account and suggest that LMDF and context-dependent forgetting can arise on the basis of different mechanisms. The inhibition account in its original form makes no predictions regarding whether LMDF should be long-lasting or not. Though the results thus do not directly speak to the account, they indicate that, to provide a full explanation of LMDF findings, the inhibition account would have to assume that the effects of inhibition are lasting. Such assumption, for instance, could be based on research on retrieval-induced forgetting—the finding that selective retrieval can result in forgetting of related items (Anderson, Bjork, & Bjork, 1994)—because, similar to LMDF, retrieval-induced forgetting has been attributed to an inhibitory mechanism (Anderson, 2003; for alternative accounts, see Jonker, Seli, & MacLeod, 2013; Raaijmakers & Jakab, 2013) and has been found to (sometimes) persist with delay (for a recent meta-analysis, see Murayama, Miyatsu, Buchli, & Storm, 2014).^1^ Naturally, on the basis of a variant of the inhibition account that assumes that inhibitory effects in LMDF are long-lasting, the prediction arises that LMDF results obtained after shorter delays should hold equally after longer delays.

An alternative explanation for long-delay LMDF results might also be based on a variant of the selective-rehearsal account of LMDF. Following a proposal by MacLeod, Dodd, Sheard, Wilson, & Bibi, (2003), rehearsal might play a critical role with prolonged retention intervals. Indeed, participants might have more time to engage in such rehearsal during longer delay intervals subsequent to encoding rather than during list-2 encoding. If this were the case and if participants engaged in rehearsal of list 1 after a remember cue but not after a forget cue, this difference in rehearsal alone might be able to account for persistent LMDF. Such a perspective would also be in line with the observed transience of context-dependent forgetting when induced by imagination or semantic generation tasks, because, in contrast to a forget cue, these tasks provide no reason to stop engaging in list-1 rehearsal during a delay.

## The present study

The present study focused on two core findings in the LMDF literature and examined whether they generalize from short to long retention intervals. Considering how little is currently known about LMDF after prolonged delays, understanding whether, and if so how, retention interval influences LMDF findings is of considerable empirical interest. In addition, corresponding data may help to further unravel the mechanisms mediating list-1 forgetting. Here, we focus on the selective-rehearsal and inhibition accounts, assuming that selective retrieval operates mainly during longer delays and that inhibition induces effects that are long-lasting. These variants of the accounts can lead to very different predictions.^2^

The one core finding addressed in the present study is the role of list-2 encoding for list-1 forgetting. Previous studies have shown that a forget cue per se is not sufficient to induce list-1 forgetting (Gelfand & Bjork, 1985, cited in Bjork, 1989; Pastötter & Bäuml, 2007, 2010; see also Racsmany, Demeter, & Szöllösi, 2019). In these studies, participants studied a list and received a remember or a forget cue. In both cue conditions, half the participants learned a second list; the rest completed an unrelated distractor. The forget cue induced list-1 forgetting only when learning of the second list was interpolated, indicating that list-2 encoding is a necessary condition for list-1 forgetting. Based on the variant of selective rehearsal introduced above, with delay, the provision of remember or forget cues alone should be sufficient to trigger different rehearsal activities. Thus, list-1 forgetting should no longer be limited to when a second list was encoded, but, with longer delay, may newly emerge in the absence of such list-2 encoding. In contrast, the inhibition account assumes that inhibitory processes are activated during list-2 encoding to downregulate interference from list-1 items. Therefore, inhibition should not be recruited when list-2 encoding is absent. The variant of the inhibition account that assumes lasting effects of inhibition predicts that list-1 forgetting should emerge in the presence of list-2 encoding only, and that this pattern should not vary with retention interval.

The other core finding addressed in the present study is the role of test format for list-1 forgetting. Numerous studies have shown that, after a short delay, list-1 forgetting typically arises on recall tests but is absent on tests of item recognition (e.g., Basden et al., 1993; Geiselman et al., 1983; Pastötter, Kliegl, & Bäuml, 2016; Sego, Golding, & Gottlob, 2006). The absence of list-1 forgetting in item recognition after short delays argues against a critical contribution of encoding differences to list-1 forgetting, as they should arise based on selective rehearsal. Yet, if selective rehearsal became more important with a longer delay, list-1 forgetting should newly emerge on recognition tests after such delay. In contrast, the inhibition account is consistent with the observed role of test format for list-1 forgetting, because recognition tests provide a chance for reexposure of the items, which may release the items from inhibition (e.g., Bjork & Bjork, 1996, 2003; Geiselman et al., 1983). Moreover, if effects of inhibition are assumed to be lasting, the role of test format should also not vary with retention interval and list-1 forgetting should be present on free recall but absent on item recognition after a long delay.

## Experiment 1

Experiment 1 built on previous work showing that, with a short delay between study and test, list-2 encoding is a necessary precondition for list-1 forgetting to arise (e.g., Pastötter & Bäuml, 2007, 2010). Subjects studied a first list of items and received either a remember cue or a forget cue for this list. Half of all subjects then studied a second item list, whereas the other half was distracted for the same amount of time. Following Abel and Bäuml (2019), a free-recall test on list 1 was conducted after 30 s or 20 min. For the short delay condition, we expected to replicate the previous finding that list-1 forgetting emerges in the presence but not in the absence of list-2 encoding. Expectations for the long delay conditions depend on the theoretical account. Based on the proposal that selective rehearsal becomes more important with a longer delay between study and test, the selective rehearsal account predicts that list-1 forgetting should emerge after a longer delay, irrespective of the presence or absence of list-2 encoding. In contrast, based on the proposal that inhibitory effects in LMDF are long-lasting, the inhibition account predicts that the pattern of results after a long delay should replicate the pattern after a short delay, and list-1 forgetting should emerge in the presence of list-2 encoding only.

## Method

### Participants

400 students (101 male, 299 female) at Regensburg University participated in the experiment, with 50 participants in each of eight experimental conditions. An a priori sensitivity analysis conducted with G*Power (Faul, Erdfelder, Lang, & Buchner, 2007) for between-subjects ANOVAs suggested that this sample size would be sufficient to detect small to medium-sized interaction effects of *f* = .14 (with *alpha* = .05 and 1 − *beta* = .80). Mean age was 21.40 years (*SD* = 2.55; range 18–32 years); all subjects were fluent in German. Subjects were tested individually and received a small monetary reward or partial course credit for participating.

### Material

Item material comprised two lists of 16 unrelated items, drawn from the CELEX database (Duyck, Desmet, Verbeke, & Brysbaert, 2004). Both lists contained concrete nouns commonly used in German. All nouns consisted of 1–3 syllables, with no difference in mean number of syllables across lists (list 1: *M* = 1.88 syllables, *SD* = 0.50; list 2: *M* = 1.81 syllables, *SD* = 0.40). All item materials as well as all data for the present experiments are available on the Open Science Framework (https://osf.io/c4ap5/). For all subjects, the same list was used as list-1 study material. Only half of all participants were asked to study a second list, therefore list-1 material was held constant across conditions with and without list-2 encoding. In those conditions that included list-2 encoding, the other list was used as list-2 study material.

### Design

The experiment had a 2 × 2 × 2 between-subjects design, with the factors cue (remember, forget), list-2 encoding (with, without), and delay (30 s, 20 min). After study of list 1, half of all subjects received a cue to remember the items; the other half was instructed to forget the list. Subsequently, half of all subjects in all conditions received a second list for study, whereas no second list was presented for the other half. Finally, conditions differed in whether a free-recall memory test on the studied material was conducted after a short delay of 30 s or after a longer delay of 20 min.

### Procedure

*Study phase.* Participants were asked to memorize as many of the to-be-presented words as possible. In all conditions, the 16 list-1 items were presented at a rate of 4 s per word, in random order, and centrally on a computer screen. After study of list 1, subjects in the remember-cue condition were asked to try to keep the list in mind for a later test and to count backwards from a three-digit number in steps of two for 60 s. In the forget-cue condition, a software crash was simulated and participants were told that they were accidentally presented with a corrupted file and the wrong list of words (e.g., Abel & Bäuml, 2013; Barnier et al., 2007). Subjects were asked to try to forget the list, because it would not be tested later. Delivering this cover story took about 60 s as well.

Subsequently, half of all subjects studied a second list with 16 new items. In the remember-cue condition, subjects were asked to memorize list 2 in addition to list 1, whereas, in the forget-cue condition, subjects were instructed to focus on the second list because it would be the only one relevant for a later test. After study of list 2, subjects were asked to count backwards for 30 s.

Instead of studying a second list, subjects in the no list-2 encoding condition counted backwards for 94 s (64 s to match the duration of list-2 presentation, plus 30 s that were also spent with counting backwards in the list-2 encoding condition). For subjects who had received a forget cue, this constituted the first backward-counting task. Subjects in the remember-cue condition, however, were also asked to count backwards for 60 s after list-1 study, to equate time that was spent delivering the cover story in the forget-cue condition. As a consequence, participants who received a remember cue for the first list, but were not asked to study a second list, were simply asked to count backwards for 154 s in total.^3^

Subjects in the short-delay condition immediately went on to the final test. In the long delay condition, subjects engaged in unrelated cognitive distractor tasks (i.e., the connect-the-numbers test, Oswald & Roth, 1987; the d2 test of attention, Brickenkamp & Zillmer, 1998; and moral dilemmas, Greene, Nystrom, Engell, Darley, & Cohen, 2004) for 20 min before completing the same final test.

*Test phase.* Before the recall test started, subjects in the forget cue condition were debriefed about the purpose of the computer crash and were informed that, despite earlier instructions, list 1 would indeed be relevant for the test. Recall was blocked by list to prevent differences in performance based on output interference from list-2 recall in those conditions in which a second list was encoded. All subjects started with a free-recall test on list 1 and were asked to write down as many of the presented list items as possible; they had 60 s to complete this task. Participants who had encoded a second list were subsequently asked to recall list 2 in the same manner. Analysis of memory performance was then focused on items correctly recalled on the correct list. Upon test completion, all participants were debriefed, thanked, and compensated for their participation.

## Results

### Results for list 1

**Fig. 1:**
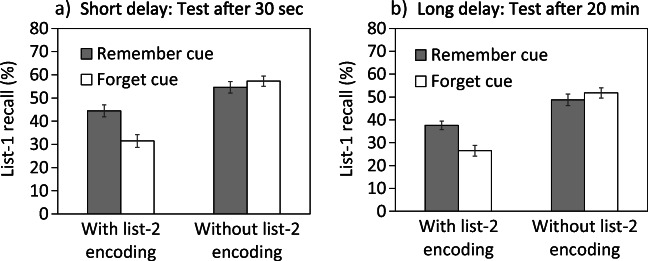
Results of Experiment 1: Mean list-1 recall as a function of cue (remember cue, forget cue) and list-2 encoding (with, without). Panel **a** shows results in the short-delay condition (30 s); panel **b** shows results in the long-delay condition (20 min). *Error bars* correspond to ± 1 standard error of the mean

Figure 1 shows mean recall rates for list-1 items in all conditions. A 2 × 2 × 2 ANOVA revealed significant main effects of cue, *F*(1,392) = 7.48, *MSE* = 286.00, *p* = .007, *η*^2^ = .02, delay, *F*(1,392) = 11.81, *MSE* = 286.00, *p* = 001, *η*^2^ = .03, and list-2 encoding, *F*(1,392) = 114.09, *MSE* = 286.00, *p* < .001, *η*^2^ = .23. List-1 recall was overall lower after a forget relative to a remember cue (41.75 vs. 46.38%), after list-2 encoding relative to no list-2 encoding (35.03 vs. 53.09%), and after longer relative to shorter delay (41.16 vs. 46.97%). Critically, there was a significant interaction between the two factors of cue and list-2 encoding, *F*(1,392) = 19.34, *MSE* = 286.00, *p* < .001, *η*^2^ = .05, suggesting that the difference in recall between cue conditions depended on the presence vs. absence of list-2 encoding. There were no further significant interactions, neither between cue and delay, *F*(1,392) = 0.11, *MSE* = 286.00, *p* = .740, *η*^2^ < .01, nor between list-2 encoding and delay, *F*(1,392) = 0.01, *MSE* = 286.00, *p* = .941, *η*^2^ < .01, or between all three factors, *F*(1,392) = 0.05, *MSE* = 286.00, *p* = .825, *η*^2^ < .01. These findings suggest that delay after study did not influence the magnitude of LMDF or the effect of list-2 encoding and, most critically, that the significant interaction between cue and list-2 encoding was not additionally modulated by delay.

For the short-delay condition, follow-up tests confirmed that list-1 recall was reduced after forget vs. remember cues in the presence of list-2 encoding (31.50 vs. 44.50%), *t*(98) = 3.44, *p* = .001, *d* = 0.69, but not in its absence (57.25 vs. 54.63%), *t*(98) = 0.79, *p* = .430, *d* = 0.16. Similar results emerged in the long-delay condition. List 1 forgetting after a forget relative to a remember cue was again only significant in the presence of list-2 encoding (26.50 vs. 37.63%), *t*(98) = 3.71, *p* < .001, *d* = 0.74, but not in its absence (51.75 vs. 48.75%), *t*(98) = 0.86, *p* = .378, *d* = 0.18. LMDF can be long-lasting, but like LMDF after short delay, the effect seems to depend critically on the presence of list-2 encoding.

### Results for list 2

Forget cues in LMDF can not only impair recall of list 1, but also enhance recall of list 2 (e.g., Bjork, 1989; MacLeod, 1998). Yet, this benefit for list 2 is often reduced or even eliminated when recall of list 1 is tested before that of list 2 (see Pastötter, Kliegl, & Bäuml, 2012), as was done in the present experiment. For completeness, mean list-2 recall is shown in Table 1. A 2 × 2 ANOVA revealed a significant main effect of delay, *F*(1,196) = 9.80, *MSE* = 390.78, *p* = .002, *η*^2^ = .05, with higher recall of list-2 items after short than after long delay (41.13 vs. 32.38%). There was no significant main effect of cue, *F*(1,196) = 0.80, *MSE* = 390.78, *p* = .372, *η*^2^ < .01, and no significant interaction between the two factors, *F*(1,196) = 0.03, *MSE* = 390.78, *p* = .858, *η*^2^ < .01, indicating that list-2 recall was not affected by cue (38.00% vs. 35.50%). These findings—with a small numerical, but no statistical difference between cue conditions—are consistent with those of previous studies.

**Table 1:** Descriptive statistics for list-2 recall in Experiment 1, shown as a function of delay (30 s, 20 min) and cue (remember cue, forget cue)

		List-2 recall in %	
Delay	Cue	M	SD
30 s	Remember	40.13	21.73
	Forget	42.13	19.18
20 min	Remember	30.88	20.22
	Forget	33.88	17.73

## Discussion

The results of Experiment 1 replicate the previous finding that, with a short retention interval, list-1 forgetting depends on post-cue encoding and arises in the presence but not the absence of list-2 encoding (e.g., Pastötter & Bäuml, 2007, 2010). The results in the long-delay condition generalize this pattern to a longer delay interval of 20 min, suggesting that the influence of list-2 encoding on list-1 forgetting does not vary with retention interval. These novel results after longer delay are not compatible with the proposal that selective rehearsal becomes more critically involved in LMDF as delay between study and test increases. Because the provision of remember or forget cues alone should be sufficient to trigger different rehearsal patterns during longer retention interval, list-1 forgetting should have emerged irrespective of list-2 encoding, which is not what the results show. Instead, the results suggest a similar pattern of list-1 forgetting after short and long delays, which is consistent with the prediction of a variant of the inhibition account that assumes that inhibitory effects in LMDF are lasting.

## Experiment 2

Experiment 1 built on previous work showing that, with a short delay between study and test, list-1 forgetting usually does not arise on standard yes/no-recognition tests (e.g., Basden et al., 1993; Geiselman et al., 1983; Sego et al. 2006). In this experiment, subjects studied two lists of items and received either a remember cue or a forget cue after the first list. Item recognition of list-1 items was tested after a brief delay of 30 s or after longer delays of 20 min or 24 h. We applied two longer delay intervals in Experiment 1 because, in contrast to recall, significant time-dependent forgetting in recognition memory sometimes only emerges after an extended delay interval.

We expected to replicate the previous finding that, after short retention interval, list-1 forgetting is absent in item recognition. On the basis of the proposal that selective rehearsal becomes particularly important with a longer retention interval, this variant of selective rehearsal predicts that list-1 forgetting emerges after a longer delay. Because no forgetting is expected after short delay in this experiment, the experiment can not serve as a direct test of the inhibition account. This holds while the inhibition account would clearly be challenged if the forgetting was found to be absent after short delay but to be present after long delay.

## Method

### Participants

An a priori sensitivity analysis conducted with G*Power (Faul et al., 2007) for between-subjects ANOVAs suggested that a sample of 312 participants would be sufficient to detect small to medium-sized interaction effects of *f* = .18 (with *alpha* = .05 and 1 − *beta* = .80). Inspection of an X-Y plot (with detectable effect sizes on the *y*-axis and sample size on the *x*-axis) further showed that high increases in sample size would be necessary to be able to detect even smaller effect sizes (e.g., a sample size of roughly 500 subjects would have been necessary to detect effects of *f* = .14). We therefore decided to recruit 312 students at Regensburg University for the experiment (266 female, 45 male, one other), with 52 participants in each of six experimental conditions. Subjects were tested individually. All participants were fluent in German and received a small monetary reward or partial course credit for participating.

### Material

There were two sets of materials, each including two lists of items. Each list consisted of 30 unrelated German nouns taken from the CELEX database (Duyck et al., 2004). For each participant, one set of materials was presented during study; the other set served as lures on the final recognition test. Across participants, each set was equally often used as study and distractor material. In addition, within sets, lists were also equally often used as list 1 and list 2.

### Design

The experiment had a 2 × 3 between-subjects design with the factors of cue (remember, forget) and delay (30 s, 20 min, 24 h). After studying list 1 and before studying list 2, participants were instructed to either remember or forget list 1. The final recognition test was carried out after 30 s, after a 20-min interval filled with unrelated cognitive tasks, or in a second experimental session after 24 h.

### Procedure

*Study phase.* Participants were presented with word lists and asked to memorize as many items as possible. All list items were presented individually and in random order for 2 s on a computer screen. Immediately after list-1 presentation, participants were cued to remember or to forget this list. In the forget-cue condition, a computer crash was simulated and subjects were asked to try to forget the list, pretending that they were accidentally presented with a wrong list, which would not be tested later. They were asked to forget the list and to memorize the following list instead, which supposedly was the correct one. Delivering this cover story took roughly 60 s. To keep timing across cue conditions constant, participants in the remember condition were asked to count backwards in steps of two for 60 s, and then were asked to memorize list 2 in addition to list 1. List 2 was then presented in the same manner as list 1.

After study of list 2, all participants counted backwards for 30 s. Participants in the short delay condition immediately moved on to the final recognition test. In the long delay conditions, the test was carried out after 20 min or after 24 h. The 20-min delay was filled with unrelated cognitive tasks, including the connect-the-numbers test (Oswald & Roth, 1987), the d2 test of attention (Brickenkamp & Zillmer, 1998), and moral dilemmas (Greene et al., 2004). In the 24-h delay condition, as a brief distraction, participants also worked on the connect-the-numbers test for roughly 5 min, but then left the lab. They were given no specific instructions on what to do during the delay, but were asked to return for a second session the next day, after 24 h. They then took the same recognition test as the other participants.

*Test phase.* Before the test began, participants in the forget condition were informed about the purpose of the cover story and that, contrary to initial instructions, list-1 items were indeed relevant for the test. At test, list 1 was always tested first, then list 2 was tested in the same way. The 30 studied list items were presented randomly intermixed with the same number of lure items. Participants were asked to decide for each individual item whether it was an old item from the study phase or a completely new item that had not been studied earlier. The test was self-paced, and participants entered their responses via the computer keyboard. Upon test completion, participants were thanked, debriefed, and compensated for their participation.

## Results

### Results for list 1

**Fig. 2:**
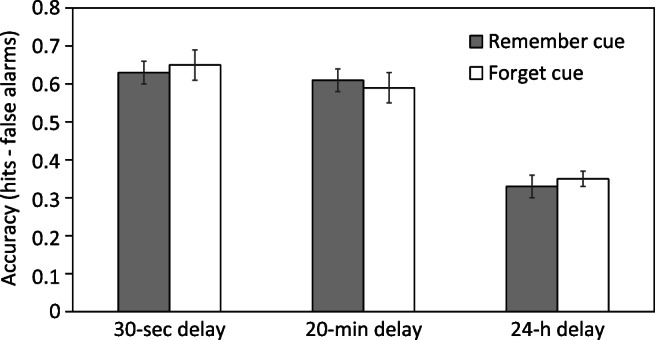
Results of Experiment 1: Mean recognition accuracy (hits minus false alarms) as a function of cue (remember cue, forget cue) and delay interval (30 s, 20 min, 24 h). *Error bars* show ± 1 standard error of the mean

Table 2 shows mean hits and false alarms. In a first step, we examined whether false alarm rates differed between forget and remember cue conditions; this was, however, not the case in any of the three delay conditions, *ts*(102) ≤ 1.52, *ps* ≥ .131, *ds* ≤ 0.30. In a second step, we then examined mean discrimination accuracy (calculated as hits minus false alarms; see also Fig. 2). A 2 × 3 ANOVA revealed neither a significant main effect of cue, *F*(1,306) = 0.01, *MSE* = 0.05, *p* = .923, *η*^2^ < .01, nor a significant interaction between cue and delay, *F*(2,306) = 0.18, *MSE* = 0.05, *p* = .837, *η*^2^ < .01, indicating that discrimination accuracy was not affected by forget vs. remember cues and that there was no LMDF in any delay condition. There was however a significant main effect of delay, *F*(2,306) = 57.09, *MSE* = 0.05, *p* < .001, *η*^2^ = .27, suggesting that discrimination accuracy was affected by retention interval between study and test. Performance did not differ between the 30 s and the 20 min delay conditions, *t*(206) = 1.21, *p* = .229, *d* = 0.17, but was significantly lower in the 24 h condition relative to both other delay conditions, *ts*(206) ≥ 8.87, *ps* < .001, *ds* ≥ 1.23.

**Table 2:** Mean hit and false alarm rates in Experiment 1, shown as a function of list (list 1, list 2), delay condition (30 s, 20 min, 24 h), and cue condition (remember cue, forget cue)

List	Delay	Cue	Hits	False	Hits - False	d$^{\prime }$
				Alarms	Alarms	
List 1	30 s	Remember	0.80	0.17	0.63	2.02
		Forget	0.78	0.13	0.65	2.11
	20 min	Remember	0.81	0.20	0.61	1.95
		Forget	0.76	0.17	0.59	1.88
	24 h	Remember	0.64	0.31	0.33	0.97
		Forget	0.63	0.29	0.34	0.99
List 2	30 s	Remember	0.73	0.21	0.52	1.57
		Forget	0.74	0.15	0.59	1.90
	20 min	Remember	0.70	0.24	0.46	1.40
		Forget	0.73	0.19	0.54	1.69
	24 h	Remember	0.60	0.32	0.28	0.82
		Forget	0.63	0.33	0.30	0.86

Another 2 × 3 ANOVA parallel to the one above was conducted with d’ as the dependent variable (calculated as z(hits) - z(false alarms); see also Table 2), but the results remained the same. There was only a significant main effect of delay, *F*(2,306) = 54.30, *MSE* = 0.66, *p* < .001, *η*^2^ = .26, but no significant main effect of cue, *F*(1,306) = 0.02, *MSE* = 0.66, *p* = .876, *η*^2^ < .01, and no significant interaction between the two factors, *F*(2,306) = 0.22, *MSE* = 0.66, *p* = .807, *η*^2^ < .01.

Because null hypothesis significance testing cannot provide support for null hypotheses (see Gallistel, 2009; Wagenmakers, 2007), we followed Masson (2011) and used the Bayesian information criterion (BIC) to compute posterior probabilities for the null and alternative hypotheses being correct given the data (D) observed in the three delay conditions. The results were remarkably similar for the three delay conditions and also across the two measures of recognition accuracy and d’, with the resulting posterior probabilities for P_*BIC*_(H_0_|D) ranging between 0.900 and 0.910, and the resulting posterior probabilities for P_*BIC*_(H_1_|D) ranging between 0.090 and 0.100. Following Raftery (1995; see also Masson, 2011), these values can be interpreted as positive evidence in favor of the null hypothesis.

### Results for list 2

For completeness, descriptive statistics for list 2 are provided in Table 2. A 2 × 3 ANOVA on discrimination accuracy (hits - false alarms) for list 2 showed a significant main effect of cue, *F*(1,306) = 4.68, *MSE* = 0.06, *p* = .031, *η*^2^ = .02, reflecting slightly higher accuracy after a forget relative to a remember cue, and a significant main effect of delay, *F*(2,306) = 34.84, *MSE* = 0.06, *p* < .001, *η*^2^ = .19, reflecting higher accuracy after short relative to prolonged delay. There was no significant interaction of cue and delay, *F*(2,306) = 0.65, *MSE* = 0.06, *p* = .524, *η*^2^ < .01. Another 2 × 3 ANOVA was conducted with d’ as the dependent variable, but the results remained the same. There was a significant main effect of cue, *F*(1,306) = 5.64, *MSE* = 0.69, *p* = .018, *η*^2^ = .02, a significant main effect of delay, *F*(2,306) = 33.67, *MSE* = 0.69, *p* < .001, *η*^2^ = .18, but no significant interaction, *F*(2,306) = 0.90, *MSE* = 0.69, *p* = .407, *η*^2^ = .01.

## Discussion

The results of Experiment 1 show no evidence for list-1 forgetting at either of the employed delay intervals. The results in the short delay condition corroborate the previous finding that, with short retention interval, list-1 forgetting is usually absent on yes/no-recognition tests (e.g., Basden et al., 1993; Geiselman et al., 1983). The results in the two long-delay conditions generalize this finding across delay intervals of up to 24 h, suggesting that list-1 forgetting may more generally be absent on such recognition tests. On a theoretical level, these results contradict predictions that arise on the basis of a variant of selective rehearsal that assumes that selective rehearsal operates mainly during longer delays. Such selective rehearsal should induce a difference in encoding, which should become evident on recognition tests when the delay is long. Regarding the inhibition account, the results can not serve as a direct test of the account, because list-1 forgetting was absent after short delay.

Although the present study had a clear focus on list-1 forgetting, the results for list 2 provided evidence for a small enhancement effect in response to the forget cue. The literature is mixed with regard to list-2 enhancement in recognition memory, with some studies failing to find benefits (e.g., Gottlob & Golding, 2007; Sahakyan, Waldum, Benjamin, & Bickett, 2009), and other studies reporting enhancement (e.g., Benjamin, 2006; Pastötter et al., 2016; Sahakyan & Delaney, 2005). Here we found list-2 enhancement, but no simultaneous list-1 forgetting, which is consistent with the view that list-1 forgetting and list-2 enhancement can arise on the basis of (partly) different mechanisms (e.g., Pastötter & Bäuml, 2010; Pastötter, Kliegl, & Bäuml, 2012; Sahakyan & Delaney, 2003). Because the main effect of cue was quite small in this experiment, conclusions regarding whether the effect in recognition memory stays the same in size or varies across delay may, however, be premature and require higher statistical power. Moreover, in the present study, list 2 was always tested last, and although there is at least some evidence that testing order may influence list-2 enhancement in recall but not in recognition (see Pastötter et al., 2016), future work may focus on examining list-2 benefits when list 2 is tested first. Results will show whether list-2 benefits reliably arise in recognition memory and whether these effects vary with delay (see also below).

## General discussion

Do factors that influence LMDF after a short retention interval still continue to do so after a longer retention interval? The present experiments show that the influence of two factors on list-1 forgetting generalizes across longer retention interval: the presence of list-2 encoding and testing format. Experiment 1 showed that list-1 forgetting depends on list-2 encoding. After both short and prolonged delay, list-1 forgetting was observed in the presence of list-2 encoding, but not in its absence. Whereas Experiment 1 found persistent list-1 forgetting in free recall, Experiment 1 found no list-1 forgetting on a standard yes/no-recognition test. Again, this pattern was the same after both short and prolonged delays. Thus, these results show that core findings from the LMDF literature can generalize from short to long retention intervals.

### Implications for the selective-rehearsal and inhibition accounts

The experiments reported here have implications regarding the potential contributions of selective rehearsal and inhibition to long-lasting LMDF. The selective-rehearsal account in its original form concerned LMDF arising after a short delay, proposing that different rehearsal patterns during list-2 encoding explain list-1 forgetting. Yet, this proposal was challenged by the early findings of short-delay list-1 forgetting with incidental encoding and eliminated short-delay list-1 forgetting on item recognition tests (e.g., Basden et al., 1993; Geiselman et al., 1983). This suggests that selective rehearsal contributes little to LMDF after a short delay. Therefore, in its original form, the account should also contribute little to explaining persistent LMDF after a longer delay.

A variant of the selective-rehearsal account, which assumes that prolonged delay after encoding provides better opportunities to engage in selective rehearsal (e.g., MacLeod et al., 2003), appears more promising. If this view held true, and selective rehearsal thus contributed little to short-delay LMDF but contributed critically to long-delay LMDF, then list-1 forgetting should increase or—in the case of no list-1 forgetting at short-delay baseline—even newly emerge when examined after long delay. The results of both experiments reported here disagree with this prediction. In Experiment 1, short-delay list-1 forgetting was absent when no second list was encoded, and critically, long-delay list-1 forgetting remained absent under this condition. In Experiment 1, short-delay list-1 forgetting was absent on yes/no-item recognition tests, and again, long-delay list-1 forgetting remained absent on this type of test. These findings challenge the idea that selective rehearsal contributes critically to long-delay list-1 forgetting.

Similar to the original version of the selective-rehearsal account, the inhibition account was also directed at explaining short-delay LMDF, leaving it open whether LMDF should be short-lived or long-lasting. Yet, as suggested by Abel and Bäuml (2019), the recent finding that list-1 forgetting can be long-lasting indicates that, to provide a full account of list-1 forgetting, the inhibition account would have to assume that inhibitory effects in LMDF are long-lasting. Importantly, with this additional assumption, the inhibition account predicts that list-1 forgetting as observed after short delay should largely generalize across prolonged retention intervals. The results of Experiment 1 are consistent with this prediction. The results of Experiment 1 can not serve as a direct test of the inhibition account, because no list-1 forgetting was present after short delay. On the other hand, the results are also not in conflict with the account, because list-1 forgetting was found to be absent after both short and long delay.

The present findings are in line with prior work by Abel and Bäuml (2019). In one of their experiments, Abel and Bäuml followed the seminal study by Geiselman et al., (1983) and applied both intentional and incidental encoding during study. The idea of the experiment was that selective rehearsal should mainly apply to intentionally studied information, but less to incidentally encoded materials. Abel and Bäuml (2019) replicated the original finding and found list-1 forgetting after a short (30 s) delay for both types of encoding. More importantly, the same pattern was also observed after a longer (20 min) delay. Similar to the present findings, these previous results thus generalize an important finding on short-delay LMDF to a prolonged retention interval. Also, like the present results, this previous result is consistent with a variant of the inhibition account that assumes long-lasting effects of inhibition in LMDF, and speaks against a critical involvement of selective rehearsal in long-lasting LMDF.

Arguably, distractor tasks as used in the present study may simply be too demanding for additional rehearsal activities, thus reducing or even eliminating selective rehearsal during the retention interval. Type of distractor activity during a 20-min delay interval was varied in another experiment reported by Abel and Bäuml (2019), the idea being that less demanding (vs. highly demanding) distractors should leave participants more mental capacity to engage in rehearsal during delay. The results, however, showed that LMDF was present after delay when the delay was filled with demanding distractor tasks but was absent after delay when the delay was filled with less demanding tasks. This finding not only disagrees with a selective rehearsal-based explanation for long-lasting LMDF, but more generally indicates that the role of distractor activity for amount of selective rehearsal may not be as straightforward as one might assume.

### Relation to the context-change account

The present experiments were not designed to provide critical tests of the context-change account of LMDF, but the results can nevertheless be considered from a context-change perspective. Experiment 1 applied yes/no-recognition tests to examine LMDF, and the results showed no list-1 forgetting after 30 s, 20 min, and 24 h. As suggested by Sahakyan et al. (2013), the fact that short-delay list-1 forgetting typically does not arise on such memory tests is in line with the context-change account, because yes/no-recognition tests may depend much less on retrieval of contextual information. The same argument can be applied to explain the absence of list-1 forgetting in recognition after a long delay. Moreover, this absence of forgetting after long delay also fits with the view that, if list-1 forgetting reflected a form of context-dependent forgetting—similar to the forgetting that arises in response to imagination or semantic generation—, it should generally be absent after long retention interval (e.g., Abel & Bäuml, 2017; Divis & Benjamin, 2014; Sahakyan et al., 2013).

The case is different for Experiment 1, which examined the role of list-2 encoding for persistent list-1 forgetting. For the short delay, the basic finding that list-1 forgetting emerges in the presence of list-2 encoding, but not in its absence, can be reconciled with the context-change account. Indeed, although arguably context change should create forgetting regardless of whether there is encoding of further material (e.g., Godden & Baddeley, 1975), a forget cue might induce weak context change only and list-2 study might then strengthen the establishment of the new mental context, which may make list-2 study necessary to observe list-1 forgetting (see Pastötter & Bäuml, 2007). More critically, Experiment 1 confirmed that, in the presence of list-2 encoding, list-1 forgetting can persist across a prolonged retention interval. This part of the results is at odds with the prediction of the context-change account that list-1 forgetting as a form of induced mental context change should dissipate with the passage of time and thus challenges the account. This holds even more as context-dependent forgetting as induced by imagination or semantic generation tasks has in fact been shown to be short-lived (Abel & Bäuml, 2017, 2019; Divis & Benjamin, 2014).

### Future directions

The finding that list-1 forgetting can be long-lasting has provided new impetus for the theoretical debate on which mechanism(s) mediate LMDF. It has created new variants of selective rehearsal and inhibition and, in the future, may also create new variants of the context-change account able to handle the persistence finding. If so, a high priority for future LMDF research may be to further examine whether patterns of short-delay LMDF generalize to long delays. Indeed, knowing whether the influence of single factors on list-1 forgetting varies with delay can impose important restrictions on theoretical accounts and may thus help to better evaluate which mechanism is most promising to explain LMDF. The present study as well as the prior work by Abel and Bäuml (2019) made some initial steps into this direction, but based on the decades-long research on short-delay LMDF there is certainly much more work to be done to draw firm conclusions on the role of delay in LMDF.

Research on LMDF during the past two decades has largely focused on list-1 forgetting, thus contrasting with the early perspective on LMDF that voluntary forgetting of outdated information (list 1) can prevent no longer needed information from interfering with relevant information (list 2; e.g., Bjork, 1970). Indeed, for short-delay LMDF, the forget cue has been shown to reduce and even eliminate interference arising from list 1 and thus to keep list-2 recall levels high (e.g., Bäuml & Kliegl, 2013; Bjork & Bjork, 1996). The fact that list-1 forgetting seems to be lasting establishes an important precondition for list-2 enhancement to persist as well. And in fact, the results of a recent study indicate that, like list-1 forgetting, list-2 enhancement may persist across longer retention intervals (Hupbach, 2018). Notice, however, that persistence of list-2 enhancement is not implied by persistence of list-1 forgetting, given that list-1 forgetting and list-2 enhancement are at least partly mediated by different cognitive mechanisms (Pastötter & Bäuml, 2010; Sahakyan & Delaney, 2003).

Indeed, two reports in the literature suggest intact list-2 enhancement after delay, even though list-1 forgetting seemed to dissipate in these studies. In the dissertation work by Liu (2001), list-1 forgetting was numerically smaller after a longer (22 min) relative to a shorter (3 min) delay interval; the critical cue x delay interaction was however not significant, making it hard to draw firm conclusions on how delay affected LMDF. Using an applied notebook-shopping scenario, Shapiro, Lindsey, and Krishnan (Shapiro et al., (2006)) reported a significant cue x delay interaction for list-1 forgetting, with intact forgetting after a shorter (3 min), but not after a longer (18 min) delay. Critically, no such interaction was observed for list-2 enhancement, which was present after both retention intervals. Shapiro et al. however used product attributes of notebooks as study materials. Moreover, in addition to providing a forget cue for a presumably outdated list of standard product attributes, participants were asked to imagine radical technological change, which made it necessary to memorize a new list of futuristic (i.e., not yet existent) product attributes. These instructions may have prompted mental context change, leaving it unclear whether the results arose on the basis of a forget cue, an imagination task that created mental context change, or both.

In any case, if both basic effects of the forget cue were present after long retention intervals (as suggested by Hupbach, 2018), then dissociations between list-1 forgetting and list-2 enhancement, as they have been observed after short delay, may represent a particularly interesting set of findings to investigate the role of delay in LMDF. Indeed, dissociations between list-1 forgetting and list-2 enhancement have been reported repeatedly in the literature. For instance, list-1 forgetting has been shown to be present without list-2 enhancement in incidental learning (Sahakyan and Delaney, 2005). Another example is the finding that number of encoded list-2 items influences list-1 forgetting and list-2 enhancement in opposing ways—with more forgetting but less enhancement when number of list-2 items increases (Pastötter & Bäuml, 2010). Knowledge on whether such dissociations survive a longer delay would critically add to the current literature on LMDF. It might motivate further tests of contemporary accounts of LMDF, and even lead to the development of more elaborated accounts.

## Conclusion

The results of the present study show that two core findings on LMDF after a short retention interval can be generalized to LMDF after a prolonged retention interval. After a longer delay, LMDF continues to be absent in item recognition, and also continues to depend on the presence of list-2 encoding. LMDF, as it has been observed after short retention intervals for decades, may thus generalize to long retention intervals. From a theoretical perspective, the results disagree with the proposal that, after prolonged delay, selective rehearsal critically contributes to LMDF. However, the results are in line with an inhibition view that assumes that inhibitory effects in LMDF are lasting. Further studies on long-delay LMDF are required, which may lead to more elaborated accounts of LMDF.

## Footnotes

1 The results regarding persistency of retrieval-induced forgetting are somewhat mixed. While some studies reported an elimination of retrieval-induced forgetting after longer delay (Abel & Bäuml, 2014; Chan, 2009; MacLeod & Macrae, 2001), other studies reported lasting forgetting (e.g., Garcia-Bajos, Migueles, & Anderson, 2009; Racsmany, Conway, & Demeter, 2010; Storm, Bjork, & Bjork, 2012; for an overview, see Bäuml & Kliegl, 2017).

2 The present experiments were designed to tease apart these different predictions, and were not designed to test the context-change account of LMDF. Nevertheless, one may consider the results also from a context-change perspective (see the General Discussion for details).

3 Counting tasks are often employed as distractor tasks in memory experiments, and they are usually highly effective in preventing rehearsal. In the present experiments, participants were also asked to count aloud, to ensure their engagement in the counting task.
